# Expedition Medicine (Revised Edition), D. Warrell and S. Anderson, eds. New York, Fitzroy Dearborn, 2003. 398 pp, hardbound. Price: $55.

**DOI:** 10.3201/eid0909.030440

**Published:** 2003-09

**Authors:** Jonathan E. Kaplan

**Affiliations:** * Centers for Disease Control and Prevention, Atlanta, Georgia, USA

Henry M. Stanley, in his second trans-Africa expedition of 1874–1877, lost 68% of his 356 men. Among the casualties, 58 died in battle or were murdered (several were cannibalized), 45 died of smallpox, 21 from dysentery, 14 drowned, and 1 was killed by a crocodile; several others died of starvation (all of this from the preface to this book). Modern-day expeditions—defined as organized and usually challenging journeys with a specific purpose of exploration, research, education, or discovery—are generally less dangerous than that experienced by Stanley. But they require extensive planning and preparation, by both leaders and expedition members, to reduce the frequencies of injury, illness, and death potentially associated with such adventures. This book is a compendium of information that will be useful to those who plan and participate in such journeys.

The editors have divided their book into three sections: expedition planning, field medicine, and specific environmental settings; each section comprises 7–14 chapters written by a total of 24 contributors. The section on planning includes advice on expedition risk assessment, assembling of medical kits, and first aid training. The second section addresses base camp hygiene, water purification, and care of various minor and serious conditions that may be encountered in the field; and the third addresses problems specific to high-altitude, polar, jungle, desert, and aquatic environments.

A major strength of this book is that, while targeted primarily to those (e.g, medical officers) who will be responsible for the health of expedition members, the writing is not highly technical. Hence, it is also suitable for paramedical personnel and other expedition members who may be interested (as they should be) in health issues specific to their expedition. In fact, this book is useful reading for those who may not have the background, time, or resources to join an expedition, but who simply enjoy wilderness experiences or ecotours for recreational purposes. As the editors point out, with the increasing availability of vacations in remote places offered by specialty tour companies, the boundary between such journeys and expeditions has become blurred. The book contains numerous tables and figures, which add to its readability. Inclusion of exotic subjects, such as treatment of bites by sea snakes and scorpions and attacks by large animals, makes for interesting reading.

The chapters vary somewhat in value to the reader. The chapter on commonly encountered ailments, such as gastrointestinal and respiratory illnesses, is very useful. The one on assessment of the injured patient is rather long; it is difficult to imagine wading through this chapter and recording various findings while managing the critically injured person in the field. The chapter on heat-related injuries fails to distinguish between heat exhaustion, heat stroke, and hyponatremia, conditions with different clinical presentations, management requirements, and prognosis. The chapter on medical aspects of survival is brief and not very useful.

Notwithstanding these minor shortcomings, this is a useful volume not only for those who plan and participate in expeditions but also for those of us who may aspire to join an expedition or who just dream of visiting exotic places. I recommend a copy for your bookshelf; better yet, for your backpack.

